# Adoptive transfer of *Trichinella spiralis-activated* macrophages can ameliorate both Th1- and Th2-activated inflammation in murine models

**DOI:** 10.1038/s41598-019-43057-1

**Published:** 2019-04-25

**Authors:** Shin Ae Kang, Mi-Kyung Park, Sang Kyun Park, Jun Ho Choi, Da In Lee, So Myong Song, Hak Sun Yu

**Affiliations:** Department of Parasitology and Tropical Medicine, School of Medicine, Pusan National University, Yangsan, Gyeongsangnam-do Republic of Korea

**Keywords:** Parasitology, Immunological disorders

## Abstract

*Trichinella spiralis* is a zoonotic nematode and food borne parasite and infection with *T*. *spiralis* leads to suppression of the host immune response and other immunopathologies. Alternative activated macrophages (M2) as well as T_reg_ cells, a target for immunomodulation by the helminth parasite, play a critical role in initiating and modulating the host immune response to parasite. The precise mechanism by which helminths modulate host immune response is not fully understood. To determine the functions of parasite-induced M2 macrophages, we compared the effects of M1 and M2 macrophages obtained from *Trichinella spiralis-*infected mice with those of *T*. *spiralis* excretory/secretory (ES) protein-treated macrophages on experimental intestinal inflammation and allergic airway inflammation. *T*. *spiralis* infection induced M2 macrophage polarization by increasing the expression of CD206, *ARG1*, and *Fizz2*. In a single application, we introduced macrophages obtained from *T*. *spiralis-*infected mice and *T*. *spiralis* ES protein-treated macrophages into mice tail veins before the induction of dextran sulfate sodium (DSS)-induced colitis, ovalbumin (OVA)-alum sensitization, and OVA challenge. Colitis severity was assessed by determining the severity of colitis symptoms, colon length, histopathologic parameters, and Th1-related inflammatory cytokine levels. Compared with the DSS-colitis group, *T*. *spiralis-*infected mice and *T*. *spiralis* ES protein-treated macrophages showed significantly lower disease activity index (DAI) at sacrifice and smaller reductions of body weight and proinflammatory cytokine level. The severity of allergic airway inflammation was assessed by determining the severity of symptoms of inflammation, airway hyperresponsiveness (AHR), differential cell counts, histopathologic parameters, and levels of Th2-related inflammatory cytokines. Severe allergic airway inflammation was induced after OVA-alum sensitization and OVA challenge, which significantly increased Th2-related cytokine levels, eosinophil infiltration, and goblet cell hyperplasia in the lung. However, these severe allergic symptoms were significantly decreased in *T. spiralis-*infected mice and *T. spiralis* ES protein-treated macrophages. Helminth infection and helminth ES proteins induce M2 macrophages. Adoptive transfer of macrophages obtained from helminth-infected mice and helminth ES protein-activated macrophages is an effective treatment for preventing and treating airway allergy in mice and is promising as a therapeutic for treating inflammatory diseases.

## Introduction

Macrophage activation can be generally divided into two distinct categories: classically activated macrophages (M1) and alternatively activated macrophages (AAM, M2). After infection with bacteria or viruses, Th1 cytokine interferon (IFN)-gamma and lipopolysaccharide (LPS) polarize macrophages toward the M1 phenotype, which induces macrophages to upregulate interleukin (IL)-12 and inducible nitric oxide synthase (iNOS) expressions. In contrast, M2 macrophages are driven by the Th2 cytokines IL-4 and IL-13. M2 cells also express high levels of arginase-1 (ARG1), mannose receptor, and resistance-like molecule-α^1–4^.

In addition, M2 macrophages can be further subdivided into the M2a, M2b, and M2c categories based on gene expression profiles. The common characteristic of all three subpopulations is the low production of IL-12 accompanied by high IL-10 production. One of their signatures is the production of enzyme Arg1, which depletes _L_-arginine to suppress T-cell responses and deprive iNOS of its substrate^5,6^. M2a macrophages are related to the Th2 immune response such as that against parasites, and they are known to be profibrotic. They secrete profibrotic factores such as insulin- like growth factor(IGF), TGF- β and fibronectin to contribute to tissue repair^7–9^. These cells also express high levels of IL-4R, FcεR, Dectin-1, CD163, CD206, CD209, and other scavenger receptors. M2b macrophages are induced upon combine exposure to IC and TLR agonist or by IL-1R agonists and express high level s of TNF superfamily 14 and CCL1. These cells express and secrete high levels of the anti-inflammatory cytokine IL-10 and low levels of IL-22, which is the function al converse of M1 macrophages. They also produce IL-1, IL-6, and tumor necrosis factor-α Base on the expression of cytokines and molecules, M2b macrophages regulate the immune response and the inflammatory reaction^8,10–12^. M2c induced by IL-10 via activating signal transducer and activator of transcription 3 (STAT3) through IL-10R and strongly exhibit anti -inflammatory activities by releasing substantial amounts of IL-10 and profibrotic activity secreting high levels of TGF- β^7,8,13^.

The regulation of macrophage function involves the activation of various transcriptional pathway-related molecules including members of the interferon-regulatory factor (IRF) family, signal transducer and activator of transcription (STAT) families, and nuclear factor-κB^14–17^. A recent study described a role for IRFs in macrophage polarization^18,19^. Many studies have demonstrated that IRF5 promotes inflammatory macrophage polarization in LPS-stimulated macrophages^20,21^. Additionally, LPS signaling through TLR4 pathways induces the activation and phosphorylation of the transcription factor IRF3, which regulates M1 gene expression^22,23^. In addition, numerous studies have shown that IRF4 promotes M2 macrophage polarization, and that expression of ARG1 was induced in IL-4-stimulated macrophages^24,25^.

For many studies, there is much interest in whether helminth-associated immune regulation may alleviate disease such as allergy and IBD^26,27^. Many helminth species have been showed to modulate allergic responses, most notably the intestinal nematode *H* . *polygyrus*, which suppresses airway hyperresponsiveness, lung histopathology, eosinophil recruitment, and Th2 cytokines in alum-sensitized models and IgE and anaphylaxis in a peanut allergy model^28–31^. Schistosome infections, egg or adult worm extract suppressed Th1/17 responses and pathology and induced the Th2 cytokines IL-10 and TGF- β^32–34^. Also, our previous study demonstrated that *Trichinella spiralis* infection derived T_reg_ cells were the key cells mediating the amelioration of allergic airway inflammation and DSS-induced colitis in mice^35,36^. Infection with parasites such as *Brugia malayi* and *Schistosoma mansoni* triggers M2 macrophages^37–41^. A previous study reported that *T. spiralis* infection induced YM1-expressing M2 macrophages, but the function of these cells remains unclear^42–44^. Evidence of immune modulation of macrophage derives from cancer models in which tumor-associated macrophages have been reported to both promote tumor survival and suppress tumor immunity. Several studies have investigated the regulatory role of macrophages in inhibiting inflammation, including models of spinal cord injury, kidney disease, and multiple sclerosis. Although these findings clearly indicate the important role of macrophages in the alleviation of inflammation^45–49^.

In this study, the functional characteristics of macrophages induced by *T. spiralis* infection in the regulation of DSS-colitis and allergic airway inflammation were examined. The ability of *T. spiralis* ES proteins to modulate macrophage activation *in vitro* was determined by detecting the production of the effector molecules iNOS, Arg1, and cytokines. In addition, the effects of ES proteins in a dextran sulfate sodium (DSS)-colitis model and an allergic airway inflammation model were investigated.

## Results

### *T. spiralis* infection induced M2 macrophage polarization

**Figure 1:**
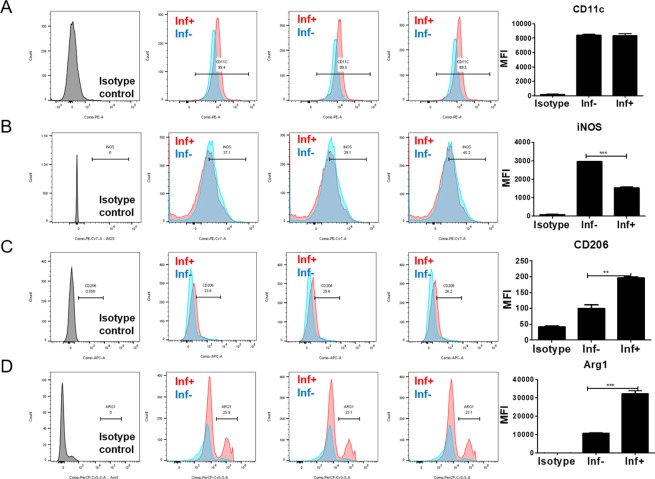
Expression of M1 and M2 markers on peritoneal macrophage. Expression levels of M1 (CD11c (**A**), iNOS (**B**) and M2 (CD206 (**C**), Arg1(**D**)) markers were analyzed in macrophages from the peritoneal macrophages (**C**) of *T. spiralis*-infected or control mice (2 weeks P.I) by using FACS canto II. After staining, the expressions of CD11c, iNOS, CD206 and Arg1 were plotted. After staining, the macrophages were firstly gated with F4/80 -positive cells and the percentage of CD11c, iNOS, CD206 and Arg1 positive cells calculated using FACS analysis. The graphs show the mean of the fluorescence intensity experimental group in the right panel. Statistical analysis was performed with Student’t t-test. (***p < 0.001; n = 3 mice/group; these results are representative of three independent experiments).

To determine which type of macrophage was activated during *T. spiralis* infection, the expression levels of M1 and M2 marker including CD11c. iNOS (M1 marker), and CD206, Argninase 1 (Arg1) (M2 marker) were evaluated in macrophages obtained from peritoneium of *T. spiralis-*infected mice at 2 weeks post infection (PI). The expression of M2 markers including CD206 and *ARG* 1 was significantly increased in of *T. spiralis-*infected mice compared to control mice at 2 weeks (PI) Whereas, the expression of M1 markers including CD11c and iNOS was not significant (Fig. 1A–D).

As a result, *T. spiralis* infection induced M2 macrophage polarization by inducing the expression of *ARG1* and *CD206* at 2 weeks (PI).

### Adoptive transfer of peritoneal macrophages from *T. spiralis*-infected mice inhibited DSS-induced colitis

**Figure 2:**
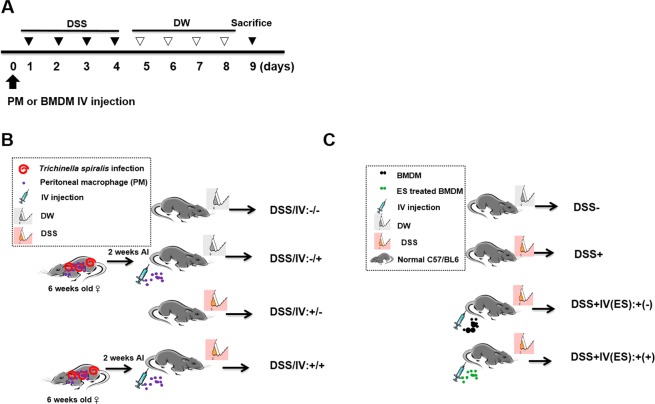
Experimental protocol of macrophage adoptive transfer and DSS-induced colitis model. Colitis was induced via DSS administration according to the experimental protocols in Methods (**A**). First, peritoneal macrophages were isolated from *T. spiralis*-infected mice at 2 weeks post-infection. The cells were stained with CellTracker CM-DiI (Life Technologies). The mice were injected intravenously with 5 × 10^5^ cells before the first DSS administration [day 0] (**B**). Second, BMDMs were isolated from C57BL/6 mice and treated with ES proteins (1 μg/mL) for 24 h. The mice were injected intravenously with 5 × 10^5^ cells before the first DSS administration [day 0] (**C**).

**Figure 3:**
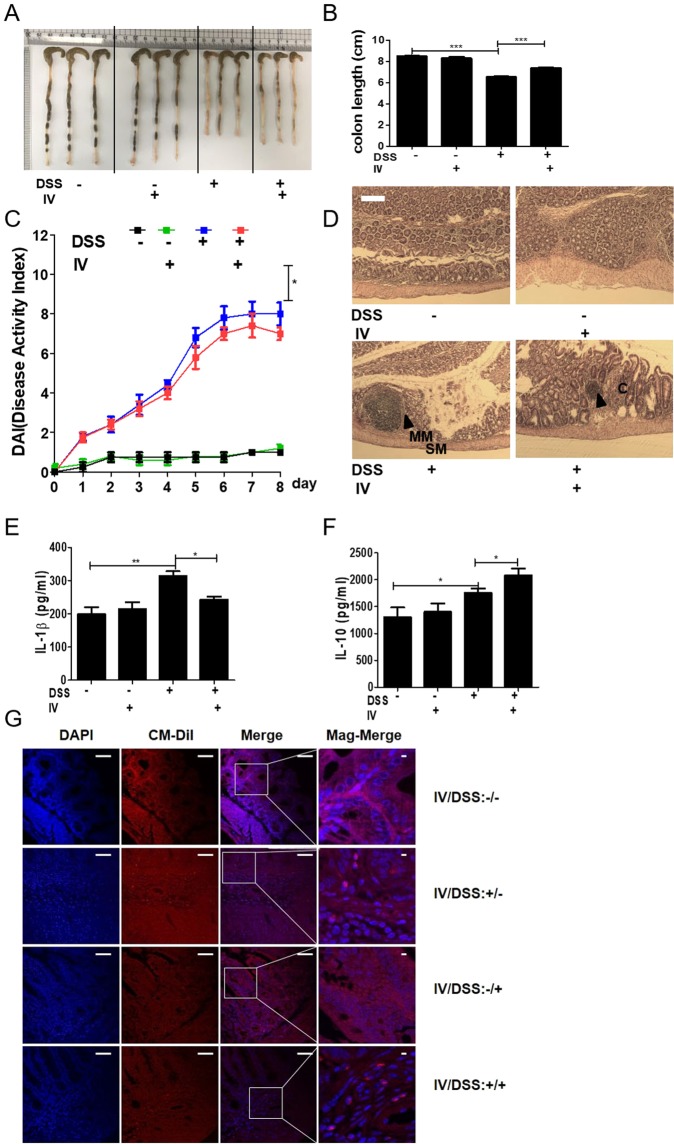
Adoptive transfer of peritoneal macrophages from *T. spiralis*-infected mice inhibited DSS-induced colitis. After sacrificing the mice, colon length was measured (**A**,**B**). The percentage weight loss, changes in stool states including consistency, and blood presence in stools of mice were examined daily during the experimental period and represented as DAI. The DAI standard is shown in Table 2. The DAI results are shown in (**C**). The large intestines were isolated from mice, rolled up, fixed with 4% paraformaldehyde, stained with H&E, and examined for histopathological changes under a microscope (**D**). Arrows indicate sub-mucosal thickening and immune cell infiltration, characteristic of colitis. The immune cells of MLN were incubated for 72 h with anti-CD3 antibody (0.5 μg/mL) for T-cell stimulation. After incubation, cytokine production in the supernatant was determined by ELISA (E, F). Large intestine sections from mice receiving peritoneal macrophages (CM-Dil, red) were immunofluorescently stained for cell nuclei (DAPI, blue). Representative pictures are shown. White bar: 100 μm (G) Statistical analysis was performed with one-way ANOVA ((*p < 0.05, ***p < 0.001; n = 3 to 5 mice/group; these results are representative of three independent experiments).

To ascertain whether *T. spiralis*-induced macrophages can prevent intestinal inflammation in the DSS-colitis model, the mice were injected with peritoneal macrophages isolated from *T. spiralis*-infected mice and assessed for intestinal inflammation responses after DSS administration. Figure Fig. 2A,B shows the experimental protocol of macrophage adoptive transfer and the DSS-colitis model. DSS administration to mice induced an acute histological change in the colon and pronounced weight loss. DSS-treated mice exhibited significant colon shortening, but the large intestines of the peritoneal macrophage-injected mice were longer than those of DSS-treated mice (6.53 ± 0.11 *vs* . 7.4 ± 0.1, *p* < 0.001; Fig. 3A,B). DSS-treated mice showed pronounced weight loss, with diarrhea and bloody stool and increased DAI from days 3 to 8. However, these clinical symptoms of DSS-treated mice after peritoneal macrophage injection were less minimized or profound compared to those of the peritoneal macrophage non-injected group at sacrifice (8 ± 1.15 *vs*. 6.75 ± 0.5, *p* < 0.05; Fig. 3C). Figure 3D shows the histological changes in the large intestine after DSS treatment. The epithelium and submucosa of the colons in DSS-untreated mice were maintained. However, the submucosa of the colon in DSS-treated mice showed immune cell infiltration, villi destruction, and ulcerative mucosa in the epithelium. While most of the epithelium remained intact, ulcerative lesions and inflammatory cell recruitment were rarely detected, and the degree of colon destruction was lower in DSS-treated mice after peritoneal macrophage injection than in DSS-treated mice. To characterize the manner in which peritoneal macrophages of *T. spiralis*-infected mice affect cytokine production from the MLN, the production of various cytokines was measured by ELISA. Peritoneal macrophage injection significantly reduced the secretion of IL-1β (314 ± 24.2 *vs*. 243.3 ± 15.27, *p* < 0.05; Fig. 3E), whereas the reduction of IFN-γ was not significant (data not shown). Furthermore, IL-10 secretion significantly increased (1757.5 ± 155.2 *vs*. 2082.5 ± 303.04, *p* < 0.05; Fig. 3F). These results indicate that *T. spiralis*-induced macrophages can inhibit the production of proinflammatory cytokines, which is consistent with the ameliorated DSS-induced colitis observed in *T. spiralis*-induced macrophage-transferred mice.

**Table 2:** Scoring system for the disease activity index (DAI).

Score	Weight loss (%)	Stool consistency	Blood
1	1~3	Normal	Negative hemoccult
2	3~6	Soft but still formed	Positive hemoccult
3	6~9	Very soft	Blood traces in stool visible
4	>9	Diarrhea	Rectal bleeding

### Transferred peritoneal macrophages infiltrate the site of inflammation

Peritoneal macrophages of DSS-colitis induction were stained with Cm-DiI fluorescent carbocyanite. Interestingly, peritoneal macrophages in the large intestine were detected in peritoneal macrophage-transferred mice. In mice without DSS administration, few peritoneal macrophages were detected in the large intestine. However, numerous peritoneal macrophages were detected in DSS-colitis induced mice (Fig. 3G). These data reveal that macrophages induced with *T. spiralis* can regulate intestinal inflammation via migration to inflamed tissues, activation, and regulation.

### Adoptive transfer of peritoneal macrophages from *T. spiralis*-infected mice inhibited airway inflammation

**Figure 4:**
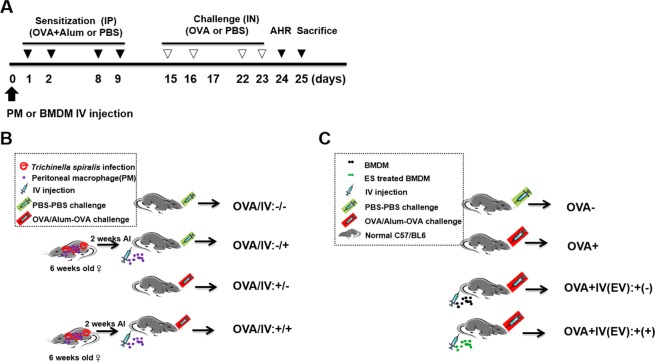
Experimental protocol of macrophage adoptive transfer and airway inflammation induction model. Airway inflammation was induced via OVA-alum administration according to the protocols in Methods (**A**). First, peritoneal macrophages were isolated from *T. spiralis*-infected mice at 2 weeks post-infection. The cells were stained with CellTracker CM-DiI (Life Technologies). The mice were injected intravenously with 5 × 10^5^ cells before the first OVA-alum sensitization [day 0] (**B**). Second, BMDMs were isolated from C57BL/6 mice and treated with ES proteins (1 μg/mL) for 24 h. The mice were injected intravenously with 5 × 10^5^ cells before OVA-alum sensitization [day 0] (**C**).

**Figure 5:**
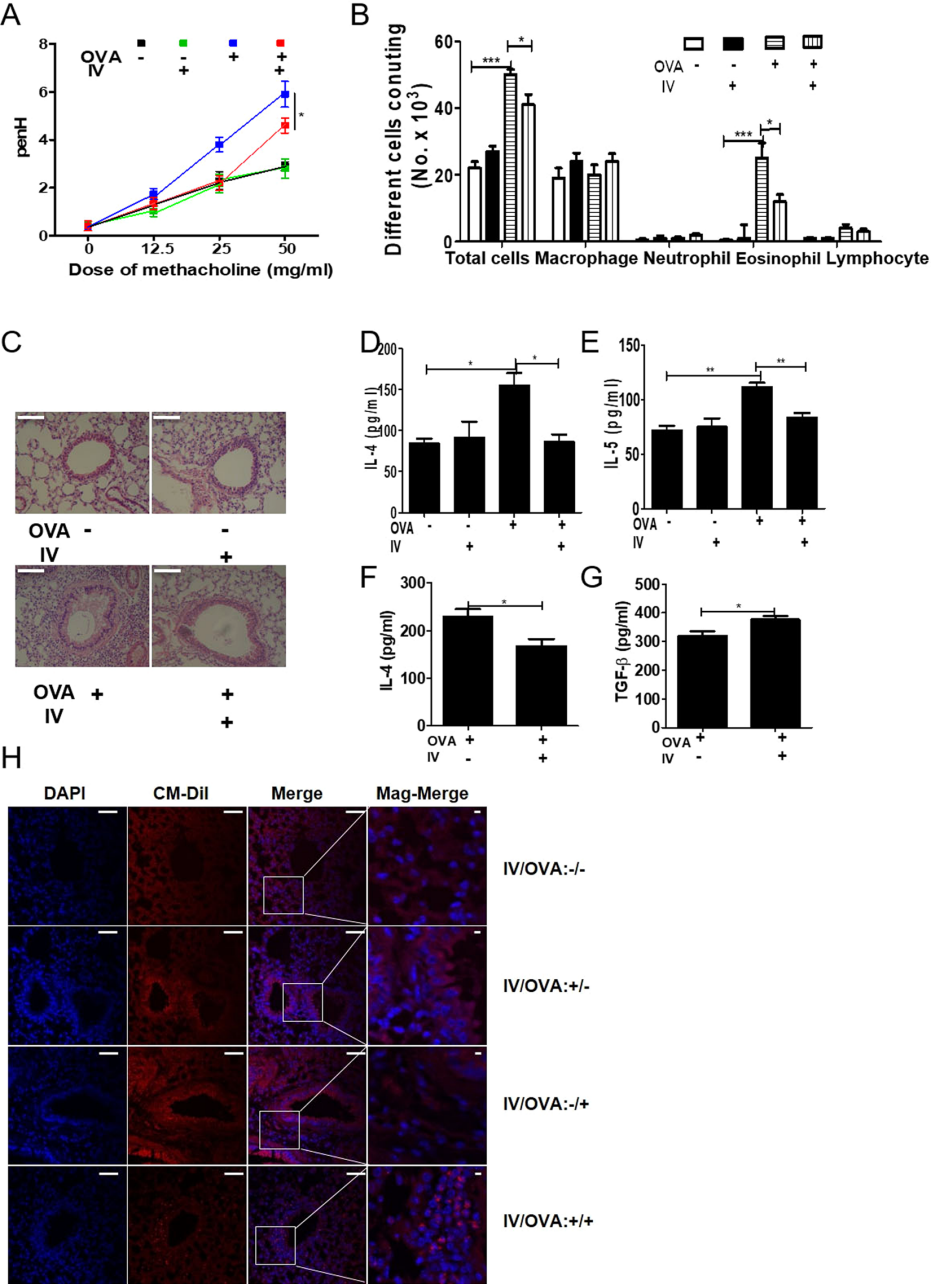
Amelioration of airway inflammation by peritoneal macrophage transfer before asthma induction. PenH was determined at baseline and after administration with increasing doses of aerosolized methacholine (0, 12.5, 25, 50 mg/mL) at day 24 (**A**). All animals were sacrificed on day 25. After sacrificing the mice, the number of differential immune cells in the BALF was counted after Diff-Quik staining (**B**). The lungs were isolated from mice, fixed with 4% paraformaldehyde, stained with H&E, and examined for histopathological changes under a microscope. Cytokine production was measured in lymphocytes isolated from the BALF (**D**,**E**) and LLN (**F**,**G**) by ELISA. Lung sections from mice receiving peritoneal macrophages (CM-DiI, red) were immunofluorescently stained for cell nuclei (DAPI, blue). Representative pictures are shown (**H)**. White bar: 100 μm. Statistical analysis was performed with one-way ANOVA and Student’t t-test (*p < 0.05, ***p < 0.001; n = 3–5 mice/group; these results are representative of three independent experiments).

To evaluate whether *T. spiralis*-induced macrophages can inhibit airway inflammation in the OVA-alum model, mice were injected with peritoneal macrophages isolated from *T. spiralis*-infected mice and intestinal inflammation responses were examined after OVA-alum induction. Figure 4 and B shows the experimental protocol of macrophage adoptive transfer and airway inflammation. PenH was used an indicator of lung function. Mice were challenged with increasing methacholine aerosols (0, 12.5, 25 and 50 mg/ml). Methacholine dose-dependently increased the value of PenH in the OVA-alum induction group. Pretreatment with *T. spiralis*-induced macrophages decreased the PenH value in OVA-alum induction (5.9 ± 1.2 *vs*. 4.6 ± 0.7, *p* < 0.05; Fig. 5A).

Different counts of cells in the BALF revealed that pretreatment with *T. spiralis*-induced macrophages before OVA sensitization and challenge significantly decreased the number of total cells and eosinophils in the BALF compared to that in mice treated with OVA only (50,000 ± 160 *vs*. 41,000 ± 300, *p* < 0.05, 25,000 ± 450 *vs*. 12,000 ± 210, *p* < 0.05, respectively; Fig. 5B). After pretreatment with *T. spiralis*-induced macrophages before OVA treatment, tissue inflammation after OVA challenge markedly decreased, and significantly less peribronchial and perivascular cellular infiltration was observed compared to that in OVA-treated mice (Fig. 5C).

To characterize the manner in which peritoneal macrophages of *T. spiralis*-infected mice affect cytokine production from BALF and LLN, the production of various cytokines was measured by ELISA. In BALF, peritoneal macrophage injection resulted in significant reductions of Th2 cytokines such as IL-4 and IL-5 (155 ± 21.2 *vs*. 86.6 ± 15.27, *p* < 0.05, 111.3 ± 7.5 *vs*. 83.3 ± 9.1, *p* < 0.01, respectively; Fig. 5D,E). In the LLN, peritoneal macrophage injection significantly reduced the secretion of Th2 cytokines such as IL-4 (230 ± 26.4 *vs*. 167.5 ± 30.9, *p* < 0.05; Fig. 5F), whereas the concentrations of TGF-β significantly increased (320 ± 26.4 *vs*. 376 ± 20.8, *p* < 0.05; Fig. 5G). Thus, these results indicate that *T. spiralis*-induced macrophages inhibit the production of Th2 cytokines, which is consistent with the ameliorated airway inflammation observed in *T. spiralis*-induced macrophage-transferred mice.

### Transferred peritoneal macrophages infiltrate the site of allergic airway inflammation

Peritoneal macrophages were stained with Cm-DiI fluorescent carbocyanite before airway inflammation. Interestingly, peritoneal macrophages in the lung were detected in peritoneal macrophage-transferred mice. Numerous peritoneal macrophages were detected in the lung of airway inflammation–induced mice (Fig. 5H). These data demonstrate that *T. spiralis*-induced macrophages can regulate airway allergic responses by migrating to inflamed tissues.

### *T. spiralis* ES proteins induced M2 macrophages

**Figure 6:**
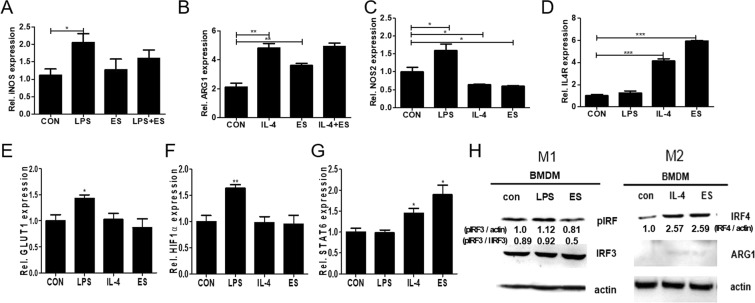
Effects of ES proteins from *T. spiralis* on M1 and M2 marker expressions in peritoneal macrophages. Primary macrophages were derived from cells in the peritoneal cavity and were cultured for 24 h in media. The gene expression levels of M1 and M2 markers were analyzed via real-time PCR (**A**–**G**). CON, cell culture medium; LPS, LPS (100 μg/mL) treatment; ES, ES proteins (1 μg/mL) treatment; LPS + ES, LPS and ES treatment; IL-4, IL-4 (20 ng/mL) treatment; IL-4+ ES, IL-4 and ES treatment. LPS and IL-4 served as M1 and M2 positive control. The cell lysates were subjected to immunoblot analysis with antibodies specific for phosphorylated forms of IRF3 and total forms of IRF4 and ARG1. The blot was re-probed with an antibody to β-actin as a control. These figures are representative of three independent experiments (areas of the detected bands were determined and compared by using Image J software (NIH, Bethesda, MD, USA) Statistical analysis was performed with Student’t t-test (*p < 0.05, **p < 0.01, ***p < 0.001; n = 3–5 mice/group; these results are representative of three independent experiments).

To investigate the effect of ES proteins on macrophage activation, we evaluated the mRNA expression of genes in BMDM cultured with or without ES proteins for 24 h. Cells were left untreated or were treated with ES proteins (1 μg/mL) for 24 h before stimulation with LPS (100 ng/mL) or IL-4 (20 ng/mL) for 1 h. As shown in Fig. 6A, ES proteins suppressed the mRNA level of M1 markers in LPS-stimulated macrophages (M1). Additionally, ES proteins alone increased the mRNA level of M2 markers in BMDM (2.12 ± 0.45 *vs*. 3.6 ± 0.24, *p* < 0.01; Fig. 6B).

To distinguish classical from alternative macrophage activation in BMDM, the gene expression levels of markers were examined. The expression of *NOS2* (nitric oxide synthase) increased in LPS-treated macrophages (1.01 ± 0.219 *vs*. 1.6 ± 0.3, *p* < 0.001; Fig. 6C). Treatment with ES proteins significantly decreased expression of *NOS2* compared to that in the control (1.01 ± 0.219 *vs*. 0.6 ± 0.0.32, *p* < 0.05; Fig. 6C). The mRNA expression of *Il4ra* (IL-4Rα) observed in IL-4-treated BMDM was significantly increased (1.01 ± 0.219 *vs*. 4.136 ± 0.059, *p* < 0.001). Treatment with ES proteins remarkably increased this expression (1.01 ± 0.219 *vs*. 5.95 ± 0.059, *p* < 0.001; Fig. 6D). The expressions of these two markers were markedly increased in ES protein-treated macrophages, suggesting alternative activation of macrophages.

Upon LPS stimulation of TLR4, a range of metabolic changes occurs in macrophages. LPS activates mTOR, thereby increasing translation of mRNA with 5′-TOP sequences, including hypoxia-inducible factor (HIF)-1α mRNA; subsequently, HIF-1α increases the expression of glucose transporter 1 (GLUT1)^50^. In this study, the expressions of genes related to metabolic regulation were measured (Fig. 6E–G). Treatment of BMDM with LPS increased the expression of GLUT1 and HIF1-α; however, IL-4 and ES protein treatments did not affect the expression of these two genes. In addition, numerous studies have indicated that strong STAT6 activation results in M2 macrophage polarization, which is associated with immune suppression^51^. The mRNA expression of STAT6 was significantly increased in the presence of IL-4 compared to that in the control. Interestingly, treatment with ES proteins significantly increased the expression of STAT6 by more than that following treatment with IL-4 (1 ± 0.165 *vs*. 1.89 ± 0.4, *p* < 0.05).

To determine whether phosphorylation of IRF3 were related to the suppression of LPS-induced proinflammatory cytokines by ES proteins, the phosphorylation of IRF3 were examined in LPS-induced macrophages by western blotting (Fig. 6H). The results showed that the levels of IRF3 phosphorylation were remarkably increased by LPS stimulation. However, treatment with ES proteins dramatically decreased phosphorylation (ratio of phosphorylated IRF3 to the total IRF3 (pIRF3:IRF3) 0.92 vs 0.57). Additionally, the expression levels of IRF4 were greatly increased by IL-4 stimulation. Treatment with ES proteins considerably increased the expression of IRF4. The expression of *ARG1* was slightly increased by IL-4 stimulation. Treatment with ES proteins slightly increased the expression of *ARG1* compared to that in untreated macrophages. The results demonstrate that ES protein treatment increased IRF4 and STAT6 expression related to M2 macrophages polarization.

### Naïve T cells with BMDMs stimulated by ES proteins induced anti-inflammatory cytokines

**Figure 7:**
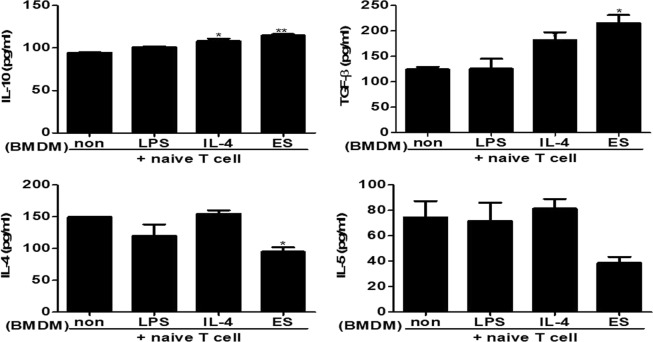
Naïve T cells with BMDM stimulated with ES proteins induced anti-inflammatory cytokines, not Th2 cytokines . Naïve T cells were cultured with BMDM stimulated by ES proteins, IL-4, or LPS or with non-stimulated BMDMs from mice for 3 days. Subsequently, the cells were incubated in the presence of anti-CD3 antibody. Expression levels of cytokines were determined in cultured supernatant by ELISA (Non: naïve T cells with non-stimulated BMDMs. LPS: naïve T cells with BMDMs stimulated with LPS. IL-4: naïve T cells with BMDMs stimulated with IL-4. ES: naïve T cells with BMDMs stimulated with ES proteins). LPS and IL-4 served as M1 and M2 positive control. (*p < 0.05, **p < 0.01; n = 4, these results are representative of three independent experiments).

ES protein-activated BMDMs from mice were co-cultivated with naïve T cells (CD4^+^CD25^−^CD62L^+^ T cells) for 72 h. After co-culture, the expression levels of cytokines in the culture supernatant were evaluated by ELISA (Fig. 7). Naïve T cells with BMDMs stimulated with ES proteins as well as IL-4 induced the production of IL-10 and TGF-β. Naïve T cells co-cultivated with ES protein stimulated macrophages did not affect the production of IFN-γ and IL-17A (data not shown). The production of Th2 cytokines, such as IL-4 and IL-5, was strongly suppressed in the supernatant of naive T cells with BMDMs stimulated with ES proteins. As a result, naive T cells with BMDMs stimulated with ES proteins increased the production of anti-inflammatory cytokines such as TGF-β and IL-10, whereas the production of Th2 cytokines was suppressed.

### Adoptive transfer of *T. spiralis* ES protein-induced macrophages inhibited DSS-induced colitis

**Figure 8:**
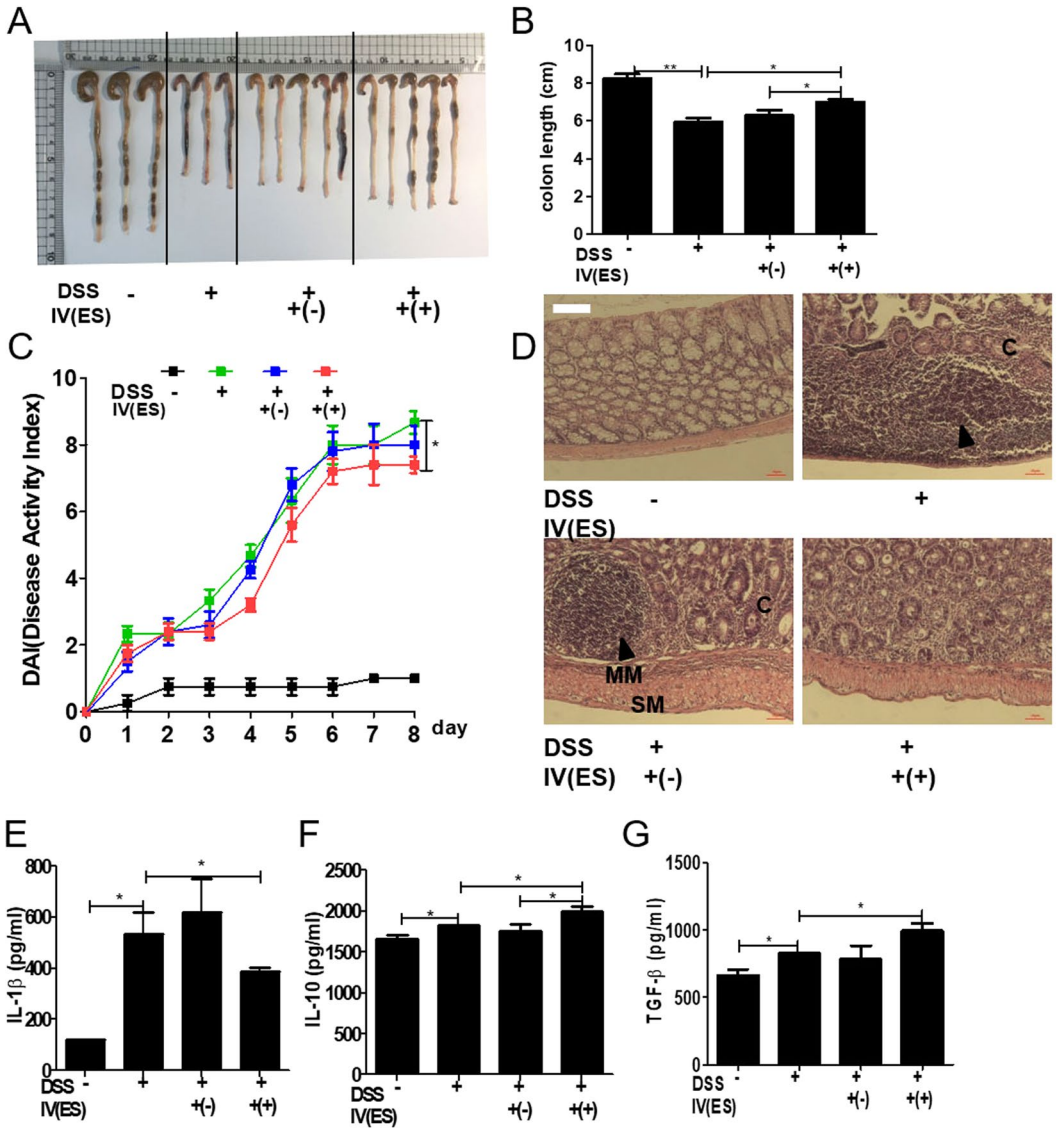
Adoptive transfer of *T. spiralis* ES protein-induced macrophages inhibited DSS-induced colitis. After sacrificing the mice, colon length was measured (**A**,**B**). The percentage of weight loss, changes in stool states including consistency, and blood presence in the stool of mice were determined daily during the experimental period and represented as DAI. The DAI standard is shown in Table 2. The DAI results are shown in (**C**). The large intestines were isolated from mice, rolled up, fixed with 4% paraformaldehyde, stained with H&E, and their histopathological changes were examined under a microscope (**D**). Arrows indicate sub-mucosal thickening and immune cell infiltration, characteristic of colitis. The immune cells of MLN were incubated for 72 h with anti-CD3 antibody (0.5 μg/mL) for T-cell stimulation. After incubation, cytokine production levels in the supernatant were determined by ELISA (**E**–**G**). Representative pictures are shown. White bar: 100 μm (H) Statistical analysis was performed with one-way ANOVA. (*p < 0.05, ***p < 0.001; n = 3–5 mice/group; these results are representative of three independent experiments).

To evaluate whether *T. spiralis* ES protein-induced macrophages can prevent intestinal inflammation in a DSS-colitis model, the mice were injected with *T. spiralis* ES protein-induced macrophages and intestinal inflammation responses were evaluated after DSS administration. Figure 2A,C show the experimental protocol for macrophage adoptive transfer and the DSS-colitis model. DSS administration to mice induced acute inflammation in the colon and caused marked weight loss and bloody diarrhea. DSS-treated mice exhibited significant shortening of the colon (8.26 ± 0.4 *vs*. 5.93 ± 0.4, *p* < 0.01), but the large intestines of *T. spiralis* ES protein-induced macrophage-injected mice were longer than those of DSS-treated mice (5.93 ± 0.4 *vs*. 7.02 ± 0.27, *p* < 0.05; Fig. 8A,B). DSS-treated mice showed continuing weight loss, with diarrhea and blood in the stool, and increased DAI scores from 1 to 8 days. However, these symptoms in DSS-treated mice after *T. spiralis* ES protein-induced macrophage injection were minimized compared to those in the *T. spiralis* ES protein-induced macrophage non-injected group (8.6 ± 0.5 *vs*. 7.4 ± 0.5, *p* < 0.05; Fig. 8C). Figure 8D shows the histological changes in the large intestine after DSS treatment. The epithelium and a sub-mucosal layer of the colons of DSS-untreated mice remained intact. However, the sub-mucosal layers of the colon in DSS-treated mice showed villi destruction and inflammatory cell infiltration as well as ulcerative mucosa in the epithelium. While most of the epithelium was intact, ulcerative lesions and inflammatory cell recruitment were rarely detected, and the degree of colon destruction was lower in DSS-treated mice after *T. spiralis* ES protein-induced macrophage injection than that in DSS-treated mice. To characterize how *T. spiralis* ES protein-induced macrophages affect cytokine production in the MLN, various cytokines were measured by ELISA. *T. spiralis* ES protein-induced macrophage injection resulted in a significant reduction in IL-1β secretion compared to that in the DSS-treated group (DSS-treated only; 535.4 ± 144.7 *vs*. 388.6 ± 25.7, *p* < 0.05; Fig. 8E) but IL-1β showed no significant reduction (data not shown). Furthermore, the secretion of IL-10 and TGF-β significantly increased (1828.6 ± 4.89 *vs*. 1195.9 ± 109.7, *p* < 0.05; 995.9 ± 4.89 *vs*. 1195.9 ± 109.7, *p* < 0.05, respectively; Fig. 8F,G). These results indicate that *T. spiralis* ES protein-induced macrophages inhibit the production of proinflammatory cytokines, which is consistent with the ameliorated DSS-induced colitis observed in *T. spiralis*-induced macrophage-transferred mice.

### Adoptive transfer of *T. spiralis* ES protein*-*induced macrophages inhibited airway inflammation

**Figure 9:**
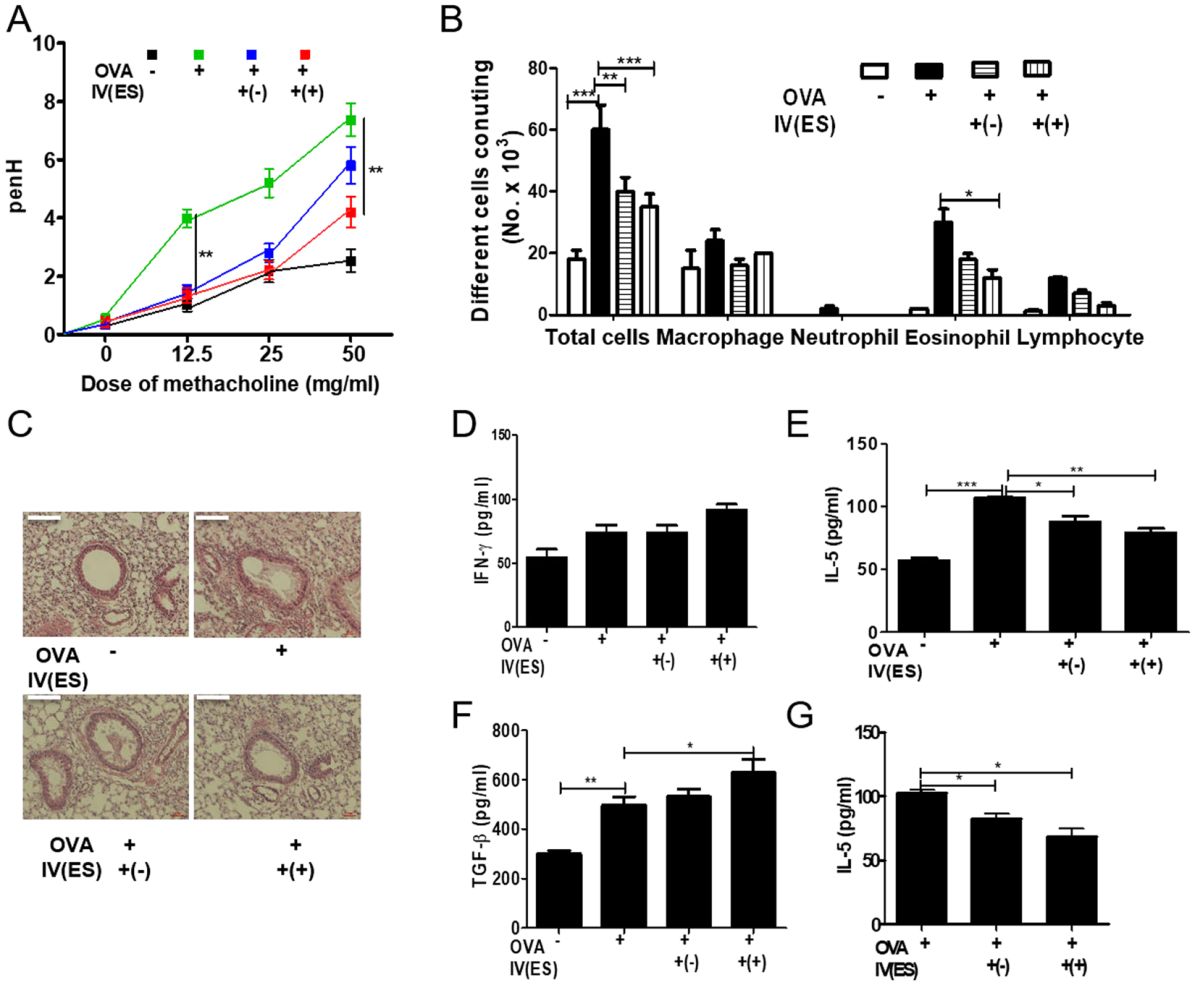
Amelioration of airway inflammation by *T. spiralis* ES protein*-*induced macrophage transfer before asthma induction. PenH was evaluated at baseline and after administration with increasing doses of aerosolized methacholine (0, 12.5, 25, and 50 mg/mL) at day 24 (**A**). All animals were sacrificed on day 25. After sacrificing the mice, the numbers of differential immune cells in the BALF were counted after Diff-Quik staining (**B**). The lungs were isolated from mice, fixed with 4% paraformaldehyde, stained with H&E, and then examined for histopathological changes under a microscope. Cytokine production was measured in lymphocytes isolated from BALF (**D**–**F**) and LLN (**G**) by ELISA. White bar: 100 μm. Statistical analysis was performed with one-way ANOVA (*p < 0.05, ***p < 0.001; n = 3–5 mice/group; these results are representative of independent experiments).

To evaluate whether *T. spiralis* ES protein-induced macrophages can prevent airway inflammation in the OVA-alum model, mice injected with peritoneal macrophages isolated from *T. spiralis*-infected mice were examined for intestinal inflammation responses after OVA-alum induction. Figure 4A,C show the experimental protocol of macrophage adoptive transfer and the airway inflammation. To assess lung function, mice were challenged with increasing methacholine aerosols (0, 12.5, 25, and 50 mg/mL). Methacholine dose-dependently increased the value of PenH in the OVA-alum induction. Pretreatment with *T. spiralis* ES protein-induced macrophages decreased the value of PenH in OVA-alum induced mice (7.37 ± 1.2 *vs*. 4.2 ± 1.2, *p* < 0.01; Fig. 9A). Differential cell counts in the BALF revealed that pretreatment of *T. spiralis* ES protein-induced macrophages before OVA sensitization and challenge significantly decreased the numbers of total cells and eosinophils in the BALF compared to that in OVA-treated mice (60,000 ± 800 *vs*. 35,000 ± 420, *p* < 0.01; 30,000 ± 430 *vs*. 12,000 ± 260, *p* < 0.05, respectively; Fig. 9B). After pretreatment of *T. spiralis* ES protein-induced macrophages before OVA treatment, tissue inflammation after OVA challenge was greatly reduced with significantly less peribronchial and perivascular cellular infiltration compared to that in OVA-treated mice and non-treated macrophage-transferred mice.

Additionally, pretreatment with *T. spiralis* ES protein-induced macrophages suppressed mucus production and hypertrophy of goblets cells compared to that in OVA-treated mice (Fig. 9C). To characterize how *T. spiralis* ES protein-induced macrophages affect cytokine production from the BALF and LLN, productions of various cytokines were measured by ELISA. In the BALF, *T. spiralis* ES protein-induced macrophage injection significantly reduced the secretion of Th2 cytokines such as IL-4 and IL-5, whereas the secretion of IL-13 showed no significant reduction (74 ± 10.5 *vs*. 91.6 ± 7.6, *p* < 0.01; 106.6 ± 1.1 *vs*. 79.4 ± 5.9, *p* < 0.001; 497.5 ± 68.9 *vs*. 630 ± 120.4, *p* < 0.05, respectively; Fig. 9D–F). Furthermore, the secretions of TGF-β were significantly increased. In the LLN, *T. spiralis* ES protein-induced macrophage injection significantly reduced the secretion of Th2 cytokines such as IL-5 (102.5 ± 3.53 *vs*. 68.5 ± 13.02, *p* < 0.05; Fig. 9G), whereas the reductions in IL-4 secretion were not significant (data not shown). These results indicate that *T. spiralis*-induced macrophages inhibit the production of Th2 cytokines, which is consistent with the ameliorated airway inflammation observed in *T. spiralis* ES protein-induced macrophage-transferred mice.

## Discussion

During helminth infections, M2 macrophages, as well as T_reg_ cells, are expanded. These cells have been shown to induce T_reg_ cell differentiation^52,53^ and, through upregulation of ARG1, have a role in tissue repair and important immune regulation by competing for _L_-arginine and generating proline^54,55^. Many study reveals an important role of YM1+ M2 macrophages in allergic lung inflammation. M2 macrophages are present in high numbers in lungs of humans and mice with allergic lung inflammation^56–59^, but their role in this disease remains controversial.

Previous study reported that M1 and M2 macrophages play opposing roles in DSS-induced colitis^60^. M1 macrophages comprise to the pathogenesis of DSS-induced colitis by secreting pro-inflammatory cytokines and causing tissue damage^61^. In contrast, similar to our observations showing that M2 macrophages contribute to the resolution of DSS-induced colitis primarily by expressing high levels of IL-10, but low levels of pro-inflammatory cytokines^60,62,63^. It has been reported chemerin aggravates DSS- induced colitis by suppresin g M2 macrophage polarization^64^. The activation of these regulatory cells during helminth infection may be responsible for the bystander suppression of immune diseases, which has been observed in several studies^65^. The purpose of this study was to determine the role of macrophages induced by *T. spiralis* . We investigated the relationship between macrophages induced by *T. spiralis* infection and immune disease. First, the M2 macrophage population, as well as that of T_reg_ cells, increased during *T. spiralis* infection (Fig. 3). *T. spiralis*-induced macrophage-transferred mice reduced the symptoms and inflammation in DSS-colitis and airway inflammation by suppressing the increased levels of Th1 and Th2 cytokines, respectively (Figs 4 and 5).

Helminths have evolved to produce molecules that enhance immune regulation. For example, the intestinal nematode parasite *Heligmosomoides polygyrus bakeri* secretes a TGF-β-like ligand that differentiates T_reg_ cells^66–68^. Given the recent advances in understanding ES proteins in helminths, we investigated the immune modulation action of ES proteins in macrophages. Pretreatment of BMDM with *T. spiralis* ES proteins significantly suppressed the expression of *iNOS* in LPS-induced macrophages (M1). However, pretreatment of macrophages with *T. spiralis* ES proteins significantly induced the expression of *ARG1* in IL-4-induced macrophages (M2) (Fig. 6). These results suggest that ES proteins inhibit M1 marker expression in LPS-stimulated macrophages and induce M2 marker expression in IL-4-stimulated macrophages. The suppression of proinflammatory activity is involved in a reduction in the severity of inflammatory diseases in *T. spiralis* infection. These results coincide with previous results in which *T. spiralis* infection suppressed host *iNOS* expression^69–72^. Additionally, *T. spiralis* ES products regulate proinflammatory cytokines^73–75^. Macrophages treated with ES proteins induced the production of anti-inflammatory cytokines such as IL-10 and TGF-β. Similar to that observed in this study, Broadhurst *et al*. reported that M2 macrophages promote Foxp3^+^ T_reg_ cell differentiation by increasing IL-10 and TGF-β^52,76^. IRFs were originally found to regulate type I IFN expression and signal pathways. However, a recent study showed that IRF regulates dendritic cells and macrophages^77^. Particularly, IRF4 was shown to regulate M2 macrophage polarization in response to parasites or chitin in the fungal cell wall^78,79^.

*T. spiralis* ES proteins promoted IRF4 and M2-related metabolic regulation via STAT6 signaling (Fig. 6). ES products of *Fasciola hepatica* were previously reported to induce M2-like phenotypes by increasing the expression of Arg-1^80,81^. Furthermore, similar to our results, YM1-expressing M2 induction has been reported in *T. spiralis*-infected mice, but the function of these cells is not established^42,43^. To directly determine the role of ES protein-induced macrophages, macrophages treated with ES proteins were prepared and transferred into mice to prepare a mouse disease model. The results show that M2 macrophages induced by *T. spiralis* ameliorated the symptoms of DSS-colitis and inhibited the Th1 response (Fig. 8). Additionally, M2 macrophages induced by *T. spiralis* reduced the symptoms of OVA-induced allergic airway inflammation and suppressed allergic Th2 responses (Fig. 9). Our data correspond well with those of Leung *et al*., who reported that M2 macrophages inhibited colitis in a dinitrobenzene sulfonic murine colitis model^82,83^. ARG1 was originally hypothesized to promote Th2-driven inflammation^84–86^. In contrast, there are some reports indicating that ARG1-expressing macrophages inhibit chronic Th2 responses. It has also been shown that ARG1-expressing macrophages suppress Th2 inflammation and fibrosis in *Schistosoma mansoni* infection^87,88^. How do M2 macrophages regulate air way inflammation? Are they activated in the peritoneum, at peripherally located lymph nodes, or at inflammatory sites? In this study, although we could not directly evaluate M2 macrophages cell population in the lung by FACS analysis because we have technical limitation such for preparation. However, we observed many CM-DII stained cells (injected cell) around inflammation sites in the airways, but it was difficult to detect cells in the absence of inflammation (Figs 4 and 5). M2 macrophages may regulate their recruitment and activation at sites of inflammation. We also did not specifically examine the cytokine/chemokine expression by macrophages in this study, but the data suggest that recruited macrophages in inflamed tissues actively contributed to the inhibiting inflammation. Taylor *et al*., reported that rodent filarial nematode *Litomosoides sigmodontis* infection of the pleural cavity, M2 macrophages are recruited to both the draining lymph nodes and the site of infection and suppress T cell responses at both sites^89^.

Indeed, there is currently controversy regarding whether M2 macrophages are effector or regulatory populations *in vivo*. The homeostatic role of M2 macrophages also extends to protecting the host from potentially harmful inflammation. A broader question yet to be resolved is whether M2 macrophages mediate primarily immunity rather than regulation. Further studies are needed to determine the specific mechanism of expansion and suppressive function of macrophages induced during helminth infection or ES proteins. Adoptive transfer of macrophages induced by *T. spiralis* is effective for preventing DSS-colitis and allergic airway inflammation and could be a promising therapeutic approach for immune diseases.

## Methods

### Animals

Six-week-old female C57BL/6 mice were purchased from Orient Bio Inc (Seongnam, Korea). They were bred in-house at the Pusan National University specific pathogen-free animal facility and accommodated according to regulations. The animals were acclimatized at a temperature of 25 °C ± 2 °C and relative humidity of 50–60% under natural light and dark condition (12 h light-dark cycle) with standard diet for 1 week prior to experimentation.

### Parasites and preparation of ES proteins of muscle larvae

The *T. spiralis* strain (isolate code ISS623) used in this study was maintained in our laboratory via serial passage in mice. The *T. spiralis* muscle larvae were collected as previously described^90^. After euthanizing mice via the inhalation of CO_2_ in a chamber, eviscerated mouse carcasses were cut into pieces and digested in 1% pepsin-hydrochloride digestion fluid for 1 h at 37 °C. The larvae were collected from the muscle digestion solution under a microscope. ES proteins were obtained as previously reported^91^.

### Cell culture and stimulation

Bone marrow-derived macrophage cells (BMDMs) were prepared as previously described^92^. Briefly, bone marrow cells were flushed from tibiae and femurs of mice. BMDM were differentiated with macrophage colony-stimulating factor (20 ng/mL) containing in DMEM with 10% fetal bovine serum and penicillin/streptomycin over 7 days at 37 °C. To evaluate the role of the ES proteins in macrophage activation, BMDM were left untreated or were treated with 1 μg/mL ES proteins, LPS (100 ng/mL), or IL-4 (20 ng/mL) for 24 h. The cells and culture supernatants were collected and stored at −80 °C for subsequent quantitative real-time PCR. Spleen, mesenteric lymph node (MLN) and lung draining lymph node (LLN) were harvested from mice euthanized by CO_2_ inhalation. Peritoneal macrophages were obtained from peritoneal lavage. Cells were isolated as previously reported^35,36,90,93^. Single cell suspensions were prepared by forcing tissue through a fine wire mesh using a syringe plunger, which was followed by repeated pipetting in RPMI-1640 with 10% fetal bovine serum and penicillin/streptomycin. RBC depletion involved cell lysis in 3 mL ACK hypotonic lysis solution (Sigma-Aldrich).

### Flow cytometry

To investigate macrophage transition during infection, live cells were isolated from the spleen and MLN of *T. spiralis*-infected mice at several times. The cell surfaces were stained with anti-F4/80-FITC(11-4801-82), anti-CD206-APC (17-2069-41) and anti-CD11c-PE (12-0114-82). anti-iNOS –PE cyanine7 (25-5920-82), and anti-Arginase 1-PerCP-eFluor 710 (46-3697-82)(eBioscience, San Diego, CA, USA) were stained using the Intracellular Fixation and Permeabilization Buffer Set in accordance with the manufacturer’s recommendations. During sample gating, cells were first gated for macrophages. The macrophage gate determined the F4/80-positive cells. CD206, CD 11c, iNOS and s Arginase 1 expression was then determined from this gated population.

### Mouse model of DSS-colitis

A mouse model of DSS-colitis was induced as previously reported with a minor modification^36,94^. Mice in the colitis induction group received 3% (wt/vol) DSS (molecular weight, approximately 40,000; MP Biomedicals, LLC, Santa Ana, CA, USA) in drinking water for 4 days, and then received drinking water for 4 days. The mice were monitored every day for morbidity and their weights were recorded. The mice were examined for weight loss, rectal bleeding, and stool consistency. The disease activity index (DAI) was used to assess the grade of colitis as described previously^94,95^.

### Mouse model of allergic airway inflammation

A mouse model of allergic airway inflammation was induced as previously reported^35,96–98^. Briefly, mice were sensitized by an intraperitoneal injection of 75 μg of OVA (Sigma-Aldrich, St. Louis, MO, USA) in 200 μL phosphate-buffered saline (PBS) containing 10 mg/mL aluminum hydroxide (Sigma-Aldrich) on days 1, 2, 8, and 9. On days 15, 16, 22, and 23 after the initial sensitization, the mice were anesthetized by using isoflurane and challenged intranasally with 50 μg of OVA in 50 μL PBS (Fig. 1A). After the last OVA challenge, airway responsiveness was monitored for change in response to aerosolized methacholine (Sigma Chemical, USA) by using whole-body plethysmography for animals (Allmedicus, Korea). The enhanced pause (PenH) was evaluated at baseline (PBS) and after treatment with increasing doses of aerosolized methacholine (0–50 mg/mL) for obtaining measurements of bronchoconstriction. The mice were permitted to acclimate for 3 min, exposed to nebulized PBS for 10 min, and then subsequently treated in methacholine using an ultrasonic nebulizer (Omron, Japan) for 10 min. After each nebulization, the average PenH values were determined during each 3 min period. Analyses of bronchoalveolar lavage fluid (BALF) and airway responsiveness were conducted as previously reported^35^.

### Cell transfer

First, peritoneal macrophages were isolated from *T. spiralis*-infected mice at 2 weeks post-infection. The cells were stained with CellTracker CM-DiI (Life Technologies, Carlsbad, CA, USA). The mice were injected intravenously with 5 × 10^5^ cells before the first DSS administration and OVA-alum sensitization [day 0]. Second, BMDM were isolated from C57BL/6 mice and treated with ES proteins (1 μg/mL) for 24 h. And then, the mice were injected intravenously with 5 × 10^5^ cells before the first DSS administration and OVA-alum sensitization [day 0].

### ELISA

An enzyme-linked immunosorbent assay (ELISA) was performed to measure the culture supernatants of MLN, LLN, and BALF by using an ELISA kit according to the manufacturer’s instructions (eBioscience).

### Histopathology

Histological analyses were conducted as previously described^99^. Briefly, lungs were harvested from mice euthanized by CO_2_ inhalation. The left lobe of a lung and the large intestine (cecum, colon and rectum) were fixed in formaldehyde and paraffin-embedded. Thin sections of the embedded tissues were stained with hematoxylin-eosin (H&E). After staining, the stained sections were evaluated under a microscope as previously described^100^.

### Immunofluorescence and confocal microscopy

The large intestine and lung slides were rinsed in PBS and incubated with DAPI only for 2 min. Confocal images of stained tissue were obtained with an inverted microscope (Olympus, Tokyo, Japan) using the Zeiss LSM program (Jena, Germany).

### Real-time PCR

Peritoneal macrophages and BMDMs were collected with 1 mL of QIAzol (Qiagen, Hilden, Germany), and RNA extraction was conducted in accordance with the manufacturer’s protocols for transcription of 2 μg of RNA. Quantitative reverse transcription-PCR assays were performed by using AmpiGene q PCR Green Mix (Enzo Life Sciences, Farmingdale, NY, USA) in a LightCycler 96 Real-Time PCR System (Roche, Basel, Switzerland). GAPDH was used as a reference gene. The primer sequences are listed in Table 1.

**Table 1:** Primer sequences for real-time PCR.

Primer	Sequence
GAPDH-for^*^	5′-TAC CCC CAA TGT GTC CGT C - 3′
GAPDH-rev^†^	5′-AAG AGT GGG AGT TGC TGT TGA AG - 3′
ARG1-for	5′-GGG GAA AGC CAA TGA AG- 3′
ARG1-rev	5′-TGG TTG TCA GGG GAG TGT- 3′
iNOS-for	5′-CGA AAC GCT TCA CTT CCA A- 3′
iNOS -rev	5′-TGA GCC TAT ATT GCT GTG GCT- 3′
FIZZ2-for	5′-GTG TTT CCT TTT CAT CCT CGT CTC- 3′
FIZZ2 -rev	5′-CAG TGG CAA GTA TTT CCA TTC CG- 3′
NOS2-for	5′-AGC TCC TCC CAG GAC CAC AC- 3′
NOS2 -rev	5′-ACG CTG AGT ACC TCA TTG GC- 3′
IL4RA-for	5′-TGA CCT CAC AGG AAC CCA GGC - 3′
IL4RA -rev	5′-GAA CAG GCA AAA CAA CGG GAT - 3′
GLUT1-for	5′-CTG CTC AGT GTC GTC TTC- 3′
GLUT1 -rev	5′-ACC CTC TTC TTT CAT CTC C- 3′
HIF-1 ɑ-for	5′-ATG AGA GAA ATG CTT ACA CAC - 3′
HIF-1 ɑ -rev	5′-TGA GGT TGG TTA CTG TTG - 3′
STAT6-for	5′-CTG GGG TGG TTT CCT CTT G- 3′
STAT6-rev	5′-TGC CCG GTC TCA CCT AAC TA- 3′

^*^for: forward; ^†^rev: reverse.

### Western blot

Cell pellets were harvested, placed on ice, washed with PBS, and resuspended in 50 μL of PRO-PREP protein extraction solution (Intron Biotechnology, Sungnam, Korea). After incubation on ice for 30 min, the lysates were centrifuged at 13,000 rpm for 30 min at 4 °C to obtain the proteins. Protein concentrations were measured using Bradford reagent (Pierce, Rockford, IL, USA). To analyze IRF3 phosphorylation, IRF4, IRF5, and ARG1 expression, 30 μg of total cellular protein was separated by 10% SDS gel electrophoresis at 100 V for 90 min. The separated proteins were transferred onto polyvinylidene difluoride membranes (Amersham Biosciences, Amersham, UK), and the membranes blocked overnight with 5% skim milk in Tris-buffered saline containing 0.1% Triton X-100. The membranes were washed five times and incubated with anti-IRF3 (rabbit monoclonal Ab, # 4302), anti-phospho-IRF3 (rabbit polyclonal Ab# 4961), anti-IRF4 (rabbit polyclonal Ab, # 4948), or anti-beta-actin (mouse monoclonal Ab#A5441) antibodies (Santa Cruz Biotechnology, Santa Cruz, CA, USA) diluted at 1:1000 in blocking buffer for overnight at 4 °C, and then underwent detection with horseradish peroxidase-conjugated secondary anti-rabbit antibody and anti-mouse antibody were used at a 1:1000 dilution for 1 hr at room temperature. The blot for horseradish peroxidase was developed by using ECL substrate (Amersham Bioscience).

### Naïve T-cell differentiation and BMDM and naïve T-cell co-culture

Naïve T cells were isolated from the spleens and lymph nodes of C57BL/6 mice by using a CD4^+^CD62L^+^ T cells isolation kit (Miltenyi Biotec, Bergisch Gladbach, Germany). Briefly, non-CD4^+^ T cells were eliminated by incubating cells with a cocktail of biotin-conjugated antibodies and anti-biotin microbeads. Next, CD4^+^CD62L^+^ T cells were labeled with CD62L microbeads and isolated by positive selection. Isolated naive T cells were co-cultured with BMDM untreated or treated with ES proteins (1 μg/mL), LPS (100 ng/mL), or IL-4 (20 ng/mL) at a BMDM:T cell ratio of 1:5 at 37 °C for 72 h in 5% CO_2_. Subsequently, the cells were incubated with anti-CD3 antibody (1 μg/mL) for 72 h at 37 °C in 5% CO_2_. The naive T-cell differentiation rate was evaluated by flow cytometry.

### Statistical analysis

Statistical analysis was performed by using GraphPad Prism 5.0 software (GraphPad, Inc., La Jolla, CA, USA) and applying Student’s *t*-test or ANOVA. For multivariate data analysis, group differences were assessed with one-way or two-way ANOVA, followed by Bonferroni’s post hoc test. All experiments were conducted in triplicate and showed similar results. Data are expressed as the mean ± SD from each experiment.

### Ethics statement

All animal studies presented were conducted in a specific pathogen-free facility at the Institute for Laboratory Animals of the Pusan National University. The experiments were registered and approved by the Pusan National University Animal Care and Use Committee (Approval No. PNU- 2016-1110) in accordance with “The Act for the Care and Use of Laboratory Animals” of Ministry of Food and Drug Safety, Korea.

### Ethical approval and consent to participate

All experimental procedures complied with the Guidelines and Policies for Ethical and Regulatory for Animal Experiments as approved by the Institutional Animal Care and Use Committee of the Pusan National University (Approval No. PNU-2016-1110).

### Consent for publication

All co-authors gave consent for publication.

## Data Availability

All relevant data are contained within the manuscript.
